# Implementation of extreme collaboration methodology in a Capstone project course

**DOI:** 10.1038/s41598-025-16699-7

**Published:** 2025-11-13

**Authors:** Eric Forcael, Luis Cifuentes, Alberto Nope, Gonzalo Garcés, Federico Casanello, Andrés Díaz-Lantada

**Affiliations:** 1 Facultad de Ingeniería, Universidad San Sebastián, Santiago, 8420524 Chile; 2 Department of Civil and Environmental Engineering, Universidad del Bío-Bío, Concepción, 4051381 Chile; 3 College of Architecture, Universidad La Gran Colombia, Bogotá, 111711 Colombia; 4 Department of Construction Engineering and Civil Engineering Projects , Universitat Politècnica de València, Valencia, 46022 Spain; 5 Vice-Presidency for Graduate and Continuing Education, Universidad San Sebastián, 8420524 Santiago, Chile; 6 Mechanical Engineering Department, Universidad Politécnica de Madrid, Madrid, 28040 Spain

**Keywords:** Extreme collaboration, Capstone project, Engineering education, BIM, Civil engineering, Information technology

## Abstract

Civil engineers’ training requires developing competencies and skills that allow them to face the challenges of professional practice. In this context, this study explores the implementation of the Extreme Collaboration (XC) methodology in an integrative civil engineering project (Capstone project) to evaluate its impact on the development of professional competencies. By integrating the Building Information Modeling (BIM) methodology and applying Bloom’s Taxonomy, the aim is to determine whether XC contributes to bridging the gap between academic training and professional practice requirements, as established by frameworks such as ABET and CDIO. Through a detailed analysis of the collaborative processes and the results obtained, the present research demonstrated that XC represents an effective pedagogical strategy to foster complex skills, such as teamwork, problem-solving, and communication, essential in the training of civil engineers capable of facing the challenges of the 21st century.

## Introduction

To obtain a professional degree in civil engineering, many universities require the students to pass a course called “Capstone Project,” which is generally carried out in the last year of civil engineering programs^1^. The Capstone Project was created in response to industry gaps in training newly graduated engineers, who often lack the practical skills and knowledge needed to succeed in the professional field^2^. In this sense, institutions such as the Accreditation Board for Engineering and Technology (ABET) and CDIO (Conceiving - Designing - Implementing - Operating) have proposed initiatives to assess the skills and capabilities that engineering students should develop in their academic life to better cope with the professional environment.

That is why various universities around the world are teaching and reinforcing multiple teaching techniques aimed at stimulating active learning in engineering education^3,4^ to train a global quality engineer and immersed in a social context^5^.

Accordingly, the Civil Engineering Body of Knowledge (CE-BOK) prescribes the depth of knowledge, skills, and attitudes required for each civil engineer entering civil engineering practice at the professional level^6,7^ promoting the need for additional academic requirements as a prerequisite for obtaining a bachelor’s degree and, consequently, professional practice from the National Council of Examiners for Engineering and Surveying (NCEES), National Society of Professional Engineers (NSPE) and the National Academy of Engineering (NAE)^7^. Thus, the implementation of innovative didactic techniques in the training of civil engineers is becoming more and more massive^8,9^ one of which is known as “Extreme Collaboration” (XC) and its application into a Capstone Project course.

Therefore, the present study highlights the importance of evaluating the fulfillment of the competencies proposed by ABET and CDIO by implementing the XC methodology applied to students in the last year of civil engineering programs. One of the motivations that inspires the realization of this study is to apply the XC methodology to a Capstone Project course to evaluate its effect on students^10,11^. Another motivation is that this type of technique has proven to be a significant contribution to design courses, improving the development of skills and capabilities of students, especially when compared to traditional methodologies.

Finally, it should be noted that there are several advantages when implementing learning strategies with peers^12^ where the XC methodology facilitates teamwork and decision-making and, therefore, the interaction between peers and the development of the capabilities of those participants who use it^10,11,13^.

Despite the growing demand for civil engineers with comprehensive competencies, a persistent gap exists between traditional academic training and the practical collaborative skills required in professional practice. Conventional teaching methods often fail to simulate the complexity of real-world engineering projects and do not effectively foster interdisciplinary teamwork and advanced problem-solving. This study addresses this shortcoming uniquely by implementing and evaluating the XC methodology in a civil engineering Capstone project, integrating Building Information Modeling (BIM) from the perspective of Bloom’s Taxonomy. Unlike previous research that may focus on isolated aspects of collaboration or technology, this holistic and contextualized approach in a purpose-built laboratory environment enables to determine how XC, with a structured four-phase procedure, directly contributes to bridging this gap, preparing students for the challenges of the 21st century in ways that traditional methodologies fail to achieve.

## Education in civil engineering programs

Over the years, engineering education has faced significant changes in its approach, seeking to contribute to developing professionals who may face new and challenging situations and provide adequate solutions in their professional lives^14,15^. Accordingly, some institutions make specific recommendations concerning engineering education approaches. An example is the American Society for Engineering Education (ASEE), which suggests patterns that engineering education should follow to maintain the pace of advances in science and technology. Thus, experimentation and an appropriate environment for developing academic activities are the keys to improving engineering students’ competencies, where the conjunction of the Capstone Project and the XC methodology point precisely in that direction.

In traditional engineering, the teaching method is eminently inductive, starting with theories and then moving to the application of these theories. All this opposes the deductive method, where case studies are presented to the students to engage them to discover and identify the concepts behind these cases. Although both teaching methods achieve student learning, evidence shows that learning can be more effective in deductive methods^16^ where Capstone Project courses are a good example^17^.

One significant limitation in engineering training is the scarcity of faculty development programs specifically designed to equip new professors with innovative teaching methodologies that align with industry demands. Engineering curricula have undergone minimal changes over the past 30 years^18^. While disciplinary mastery among engineers entering the teaching profession is undeniable, they often lack formal training in didactics and teaching strategies that facilitate the comprehensive acquisition of technical and soft skills in their students. For example, during 2022, Heydari et al.^19^ conducted research to understand the perceptions and benefits of a professional development program designed to enhance the teaching skills of novice professors in Construction Management and Engineering (CME). They presented the academic program to 15 newly recruited professors, who attended educational sessions delivered by experienced faculty. The researchers collected participants’ personal reflections and perspectives to assess the program’s impact on their teaching and mentoring skills. The results of their study revealed areas for improvement in current CME teaching practices and confirmed the value of such initiatives in preparing graduate students for future academic roles.

On the other hand, Wu and Issa^20^ conducted research to explore the relationship between university training in BIM and students’ professional development. They used a survey that allowed them to compare the perceptions of stakeholders in both the educational and professional fields. Their findings confirmed significant progress in BIM adoption in both sectors. However, they also identified a notable gap between the rapid growth of the BIM job market and the existing incentives to motivate students to pursue a career in this area. The survey also revealed that closer and more proactive collaboration between universities and industry could be key to improving BIM training and talent acquisition in the Architecture, Engineering, and Construction (AEC) sector.

These studies demonstrate that training educators in advanced methodologies, such as XC and BIM, is critical to bridging the gap between theory and professional practice and fostering competencies like teamwork, problem-solving, and communication, which are essential for the success of future civil engineers. By highlighting how a particular curriculum and teaching approach—such as those implemented in this study—benefits from an investment in teacher pedagogy development, the relevance and contribution of this research are reinforced in preparing not only students but also educators for the challenges of modern engineering.

Currently, technological advances have allowed, in civil engineering, the development of a series of laboratory activities, such as the design of buildings and civil works, the elaboration of budgets and work schedules, and the development of projects integrated design through 3D simulation and digital twin development^13,21^. All these innovations have allowed students to perform better in applied courses such as the Capstone Project^22^.

In civil engineering education, the curriculum is continuously analyzed by various institutions, such as ABET, to form properly trained engineers who have sufficient skills to face the work environment^23^. In particular, a document formulated by ABET^24^ called “Criteria for Accrediting Engineering Programs” establishes that civil engineering education programs have to consider analyzing and solving problems, along with conducting experiments in technical areas of civil engineering and interpreting the resulting data; designing a system, component, or process in civil engineering contexts, including principles of sustainability; and explaining basic concepts in project management and leadership, among other aspects. Consequently, the Capstone Project course becomes essential in training students to face the real world^25,26^.

## Capstone project course

The Capstone Project was born in response to the industrial market, which emphasized that engineers were poorly trained in practical terms and lacked skills and knowledge for the real world^26,27^. Its objective is to give the student teamwork, communication, and leadership skills, managing to promote the student’s transition to professional practices, i.e., the individual develops a change of identity from student to professional engineer^28^.

The Capstone Project in civil engineering promotes creativity. It stimulates learning through experience due to the breadth of knowledge that must be addressed, in which students must often discover knowledge that is not necessarily found in the plan of studies or the curriculum^29^. Also, it requires knowledge of administration, planning, and scheduling of works used to prepare drawings, technical specifications, and reports required to carry out a project^30^.

### Characteristics of a capstone project

ASCE establishes a series of areas in which the Capstone Project is present, such as social sciences, critical thinking, and problem-solving^31^. This perspective considers the course a transition experience, preparing students to pass to engineering professionals.

The Capstone Project is characterized by being completed by a group of students guided by advisors and professors during the project’s development, providing them with learning opportunities and acquiring new skills in a multidisciplinary environment^32^. The academic evaluation of the project is done through final deliveries of drawings, reports, documents, presentations, and group evaluations, allowing the students to get feedback on what they have learned^29^.

On the other hand, Todd et al.^33^, Dougherty et al.^34^ and Hotaling et al.^35^ listed some challenges facing the development of a Capstone Project: (1) Develop students’ professional skills and competencies, regardless of their number and the projects to be evaluated, defining the correct number of educators; (2) Increase the complexity of the projects, bringing the student closer and closer to reality; (3) Integrate the different engineering disciplines into the same project; (4) Develop project management and planning skills in students, and (5) Motivate the student to learn through their own experience, applying prior knowledge.

## Extreme collaboration

The methodology of Extreme Collaboration (XC) was created by NASA, given the need to accelerate the design of space missions, founding the “Jet Propulsion Laboratory” (JPL) as an initiative that would allow the development of this methodology^36^. This laboratory is a technological center of NASA, responsible for developing several projects by grouping different disciplines and working together simultaneously, allowing information sharing and decision-making^37^.

This methodology began gaining strength in 1994, when a team partly made up of NASA’s JPL (Jet Propulsion Laboratory) sought to optimize this collaborative work methodology, concluding that, given the effectiveness of the work and intensity that this methodology printed to the team, it was convenient to carry out integrated design sessions of 3 h, three times a week^38^. Although this methodology requires great effort and concentration from the participants, its application allows the detection of design errors through discussions and conversations and accelerating project design processes^37,39^.

The Architecture, Engineering, and Construction (AEC) has studied the application of this methodology in the construction industry, trying to carry it out in the most reliable way possible, as applied in NASA, using, for example, sessions of no more than 3–4 h and three days a week^40^. For constructive projects, the work team recommended by the AEC consists of architects, construction engineers, and engineers of each specialty required by the project^13,41^. This methodology has two active communication networks: (a) the electronic network and (b) the human network, which allow the flow of information during the XC sessions^36^.

**Fig. 1:**
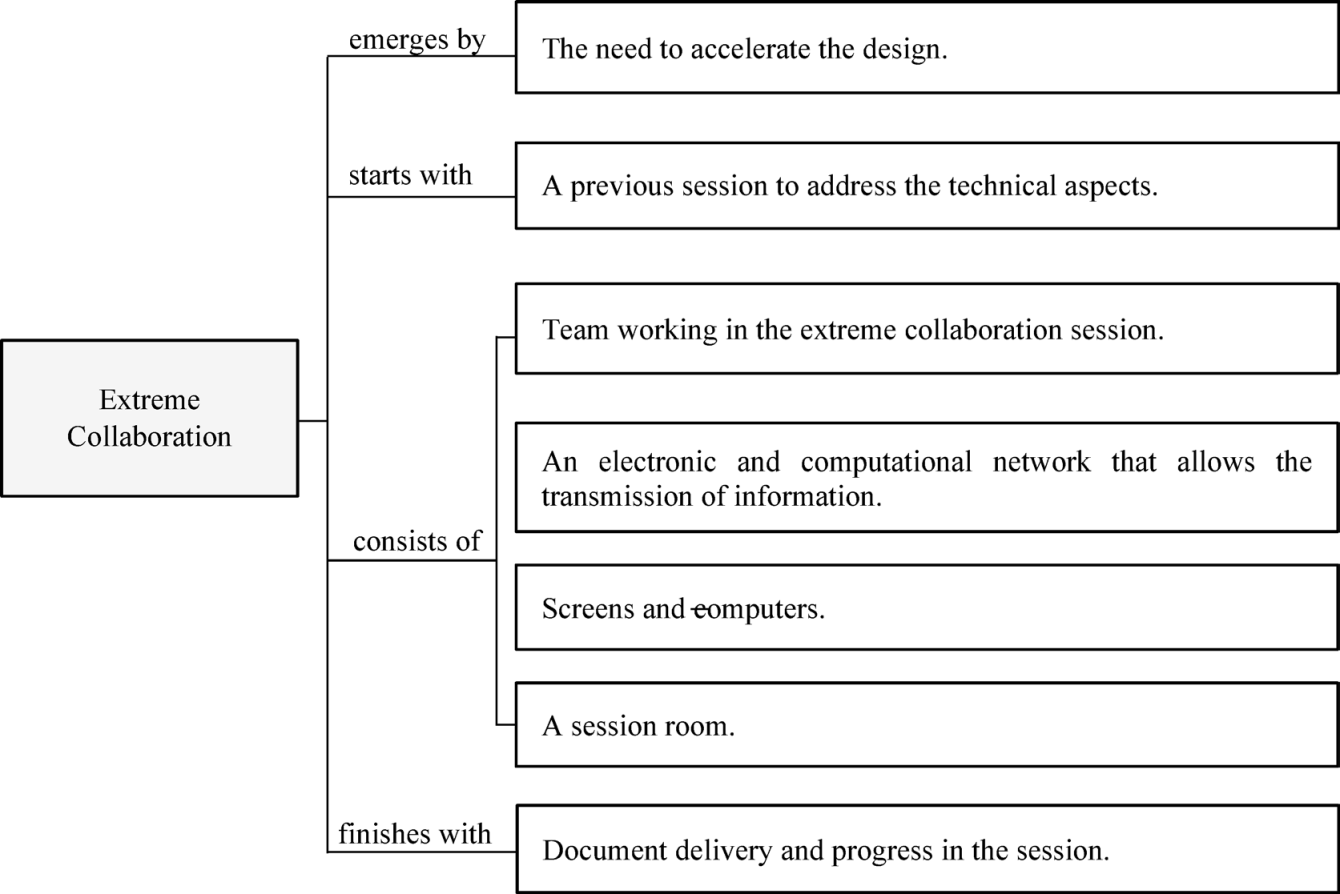
Description of the XC methodology.

The human network comprises a working team of sessions, where the participants actively create very close communications between each specialist^38^ generating multiple interactions between individuals since they continuously coordinate and adapt the information to solve the problems that arise in each of their specialties^39,42^. On the other hand, the electronic and computational network made up of the input and output devices (computers, monitors, worktables) allows the transfer of information during the sessions, keeping the participants informed of each decision taken together to update on time the calculations of each specialist^13^. The electronic network also allows the visualization and management of project data and its design instantaneously from the computer of each specialist, improving the visualization of the development and changes generated together in real time^43^. The description of the XC methodology is summarized in Fig. 1.

Some advantages of working under the XC methodology in the construction industry are the reduced time in the design stage and the optimal transmission of information among the participants^36,44^. In addition, this work methodology reduces misunderstandings and errors and resolves them at the same time they arise^45^. Because of the high standards and demands in the construction industry, the design through an XC methodology may allow advancing in the achievement of such high standards, optimizing the cost estimation, improving the quality of construction projects, detecting errors at an early stage, and minimizing the existence of poorly performed jobs^13,46^.

However, one of the limitations of working with XC is directly linked to the participants individually and collectively because this methodology requires people with flexible personalities, easy to adapt, and able to bear the burden and stress caused by conflicts in the sessions^10,47,48^. Also, they must be able to withstand the stress generated by the noise caused by the permanent interaction between the participants, along with the pressure of time, among other technical difficulties associated with 3D modeling^42^. Additionally, this methodology presents other challenges in its application, mainly related to users’ eventual lack of experience in this work modality, which could cause delays in decision-making, conflicts, and disagreements between the different specialties^36^.

## Bridging the gap: aligning capstone projects with ABET and CDIO standards through bloom’s taxonomy and extreme collaboration

Engineering education constantly evolves, requiring an ever-closer alignment between final degree projects and international standards such as ABET and CDIO. In this context, this research seeks to explore how Bloom’s Taxonomy and extreme collaboration can serve as tools to close this gap and ensure that graduates are prepared to face the challenges of 21st -century engineering.

### ABET and CDIO

The role of the engineers and their training, including professional skills, has been redefined, as well as an interest in fostering other skills and competencies, such as leadership, communication, teamwork, and ethics, within a global and social context, promoting lifelong learning and knowledge of contemporary issues^49^. In this sense, institutions such as ABET and CDIO have initiatives to assess the skills and capabilities that engineering students must develop during their academic careers to face the professional environment effectively.

CDIO’s objective is to formally summarize the knowledge, skills, and attitudes that students, the industry, and the academic world desire for future generations of engineers^50^while ABET focuses on the characteristics necessary for engineering practice to design new educational initiatives and as a basis for a rigorous evaluation process focused on results^51^. Training and preparing students in the best possible way is necessary, where initiatives such as CDIO and ABET play an essential role^8^ and new methodologies, such as the XC methodology applied to a Capstone Project course.

On the one hand, the ABET engineering criteria proposes a series of skills and attitudes every engineer should possess, including a broad knowledge of science, mathematics, and engineering^52,53^. In addition, ABET establishes a series of “soft” skills, such as communication and the ability of individuals to work as a team^54^. All these skills and attitudes are determining factors in competitiveness among engineering professionals because they allow for more appropriate development in the professional world^55,56^. This engineering approach allows the engineering student to acquire the ability to work as a team and recognize and resolve ethical conflicts, addressing each problem and getting a solution^49^.

On the other hand, engineering education seeks to give students a broad knowledge base, skills, and attitudes to turn them into successful engineers^54^. In this sense, CDIO establishes a coherent and generalizable set of objectives for engineering education^57–59^. Accordingly, CDIO argues that engineering education should be focused on teaching and transmitting skills and attitudes to get engineers that conceive, design, implement, and operate^56^ in diverse contexts and constraints^50^.

Through these outcomes, students are expected to know and do, when they graduate, the Student Outcomes (SO) and Study Program Levels (SL) shown below in Table 1, according to ABET and CDIO, respectively.

**Table 1:** Educational outcomes of students according to ABET and CDIO.

Skills and competencies of the student		
Student outcomes^a^		
ABET 2023–2024	SO1	Ability to identify, formulate, and solve complex engineering problems by applying principles of engineering, science, and mathematics
	SO2	Ability to apply engineering design to produce solutions that meet specified needs with consideration of public health, safety, and welfare, as well as global, cultural, social, environmental, and economic factors
	SO3	Ability to communicate effectively with a range of audiences
	SO4	Ability to recognize ethical and professional responsibilities in engineering situations and make informed judgments, which must consider the impact of engineering solutions in global, economic, environmental, and societal contexts
	SO5	Ability to function effectively on a team whose members together provide leadership, create a collaborative and inclusive environment, establish goals, plan tasks, and meet objectives
	SO6	Ability to develop and conduct appropriate experimentation, analyze and interpret data, and use engineering judgment to draw conclusions
	SO7	Ability to acquire and apply new knowledge as needed, using appropriate learning strategies
Syllabus levels^b^		
CDIO	SL1	Disciplinary knowledge and reasoning
		Knowledge of underlying mathematics and science Core fundamental knowledge of engineering Advanced engineering fundamental knowledge, methods, and tools
	SL2	Personal and professional skills and attributes
		Analytical reasoning and problem-solving Experimentation, investigation, and knowledge discovery System thinking Attitudes, thought, and learning Ethics, equity, and other responsibilities
	SL3	Interpersonal skills: teamwork and communication
		Teamwork and communications; and communications in foreign languages
	SL4	Conceiving, designing, implementing, and operating systems in the enterprise, societal and environmental context
		External, societal, and environmental context; and enterprise and business context Conceiving, systems engineering and management; and designing, implementing, and operating

Table adapted from^24,60–63^.

^a^SO = Student Outcomes.

^b^SL = Syllabus Levels.

To contextualize the present study, it is interesting to briefly analyze civil engineering capstone courses and similar project-based learning actions and their potential for becoming genuinely holistic educational actions. In this context, from engineering disciplines, civil engineering may arguably be the most challenging for the planning, ideation, and development of complete CDIO projects and actions. In civil engineering, the CDIO of large infrastructures like bridges, tall buildings, highways, channels, piers, and ports, among others, makes things more complicated and resource-dependent. The magnitude of the CDIO topic should not discourage colleagues because several project-based learning activities and international competitions dealing with designing and constructing innovative, efficient housing projects have been successfully carried out. The Solar Decathlon is a remarkable example^64^.

These ideas can be traced back to the Bauhaus School of Architecture, Design, Arts and Crafts, which reinvented engineering design education in Europe’s 1919–1933 period by involving students and professors in the design and construction of the actual campus building and inner houses design, constituting holistic CDIO but 80 years in advance to the foundation and the redefining of the European Area of Higher Education through the Declaration of Bologna. Other pioneering CDIO actions in civil engineering degrees include students’ projects focused on building energy, hydraulics, environmental engineering, and construction materials^65^.

The Capstone project course detailed in this study follows the spirit of such pioneering examples, concentrating on providing students with a real working scenario and experience by applying an engineering methodology validated by NASA concerning high-responsibility projects, the XC methodology.

### Bloom’s taxonomy

**Fig. 2:**
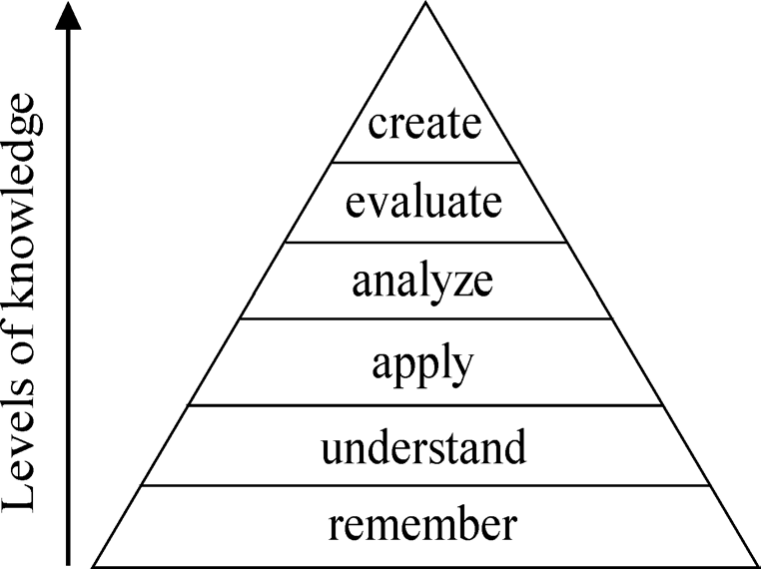
Levels Taxonomy of Bloom. (adapted from^67^.

The eight academic ambits proposed (architectural, structural modeling, etc.) were evaluated according to Bloom’s Taxonomy^66^and their different cognitive levels are shown in Fig. 2.

Bloom’s taxonomy searches for evaluating different levels of progress of the cognitive domain of the students in the learning process^68,69^going from lower to higher levels of learning and critical thinking.

On the other hand, Bloom’s taxonomy describes the progression of cognitive skills that students develop as they learn. According to^57^it begins with the most basic level, remembering, which involves recognizing and retrieving previously learned information. From there, it progresses to understanding where students demonstrate their grasp of concepts by explaining or describing them. The next level, applying, involves using acquired knowledge to solve problems or perform tasks in new situations. Analyzing involves breaking down information into parts to understand its relationships and structures. The evaluation level requires students to judge the validity or usefulness of ideas or information. Ultimately, the highest level of creation involves generating new ideas, products, or perspectives from existing knowledge. Together, these six levels provide a helpful framework for designing learning activities and assessing student progress.

Accordingly, Marzano et al.^70^ define three domain levels, which include (a) mastery of knowledge, (b) mastery of mental processes and skills, and (c) mastery of psychomotor procedures. This approach has been more used in recent years because it allows recognizing students as individuals who integrate knowledge from different ambits, offering the possibility of making projections based on the defined study object, considering as a determining aspect the use of a methodology^71,72^ such as the one used in this case, the XC methodology.

### Analysis of academic achievements using the XC methodology

The rubric presented in Table 2 is a rigorous and detailed assessment tool designed to evaluate student performance in a Capstone project developed under the Extreme Collaboration methodology using BIM. This tool is based on Bloom’s Taxonomy, a classification system for educational objectives that evaluates the complexity level of tasks and cognitive skills required.

**Table 2:** Rubric based on bloom’s taxonomy for a capstone project conducted under XC methodology.

Scope	(1) Remember	(2) Understands	(3) Apply	(4) Analyze	(5) Evaluate	(6) Create
Architectural modeling	Be able to remember the fundamental modeling process for architectural elements.	Be able to explain the practices and commands of architectural modeling.	Be able to build fundamental architectural models according to the project information.	Be able to perform an architecture analysis with the model according to the plans and specifications of the project.	Be able to evaluate the accuracy of the architecture and the integrity model against the plans and specifications.	Be able to create manufacturing and construction models based on the means and methods discussed.
Structural modeling	Be able to remember the fundamental processes of modeling structural elements.	Be able to explain the practices of the structural model and the commands.	Be able to build fundamental structural models according to the information of the planned project.	Be able to perform a structural analysis with the model according to the project specifications.	Being able to assess the accuracy of the structural model and integrity against the plans and specifications	Being able to create manufacturing and construction models based on the means and methods discussed
Sanitary-hydraulic modeling	Be able to remember fundamental processes of modeling sanitary and hydraulic elements.	Be able to explain the practices of the sanitary-hydraulic model and the commands.	Be able to build fundamental sanitary-hydraulic models according to the information of the planned project.	Be able to perform a sanitary-hydraulic analysis with the model according to the project’s specifications.	Be able to evaluate the accuracy of the sanitary-hydraulic model and the integrity of the plans and specifications.	Be able to create manufacturing and construction models based on the means and methods discussed.
Electrical modeling	Be able to remember the fundamental processes of modeling electrical elements.	Be able to explain the practices of the electric model and the commands.	Be able to build fundamental electrical models according to the information of the planned project.	Be able to perform an electrical analysis with the model according to the project specifications.	Be able to evaluate the accuracy of the electric model and the integrity of the plans and specifications.	Be able to create manufacturing and construction models based on the means and methods discussed.
Air conditioning modeling	Be able to remember the fundamental process of modeling climatic elements.	Be able to explain the practices and commands of the climate model.	Be able to build fundamental climate models according to the information of the planned project.	Be able to perform a climate analysis with the model according to the project specifications.	Be able to assess the accuracy of the climate model and integrity against the plans and specifications.	Be able to create manufacturing and construction models based on the means and methods discussed.
Budget and Cost estimation	Be able to remember the cost estimate and budget process.	Be able to explain the practices of data manipulation and cost estimation.	Be able to develop a simple budget and a budget spreadsheet.	Be able to analyze different construction systems and assemblies for pricing.	Be able to assess the costs and unit prices of each item accurately.	Be able to create models to estimate costs and budgets according to the project schedule.
Teamwork	Be able to remember critical components, roles, and responsibilities in teamwork.	Be able to explain the process of teamwork and the work of each one.	Be able to apply the structure of teamwork effectively.	Be able to analyze teamwork and identify challenges and opportunities.	Be able to evaluate the teamwork process and how it can influence the project.	Be able to create a team action plan and execute it in the engineering project.
Documentation and Manual	Be able to remember the requirements of the required documentation and the manual.	Be able to describe the documentation and the objective of the documentation format.	Be able to apply best practices for documentation and the manual.	Be able to analyze and classify different types of manuals and apply the appropriate format.	Be able to evaluate the accuracy, integrity, and professionalism of documentation and the manual.	Be able to create a manual of all the information collected and required in a professional format.

The key aspects of this rubric are:

- Alignment with Bloom’s Taxonomy: The rubric structures the assessment criteria into the six cognitive levels proposed by Bloom: remember, understand, apply, analyze, evaluate, and create. This approach ensures that everything from basic knowledge to the ability to generate innovative solutions is assessed.
- Focus on BIM-Specific Competencies: The rubric focuses on specific competencies related to BIM modeling in different disciplines (architecture, structure, sanitary, electrical, and HVAC installations), as well as on transversal skills such as teamwork, project management, and documentation.
- Integration of the Extreme Collaboration Methodology: The rubric assesses the collaborative and iterative aspects of the Extreme Collaboration methodology, evaluating the student’s ability to work as a team, adapt to changes, and make informed decisions based on available information.
- Comprehensive Project Evaluation: The rubric comprehensively evaluates the project, considering both the technical aspects (BIM modeling, calculations, etc.) and the non-technical aspects (teamwork, communication, time management).

The results obtained from applying this rubric can serve as a basis for improving teaching and learning processes in architecture and engineering. For example, the areas where students need more support can be identified, and specific learning activities can be designed to address these needs. Furthermore, the rubric can serve as a tool for individualized feedback, allowing students to identify their strengths and weaknesses, set learning goals, and analyze whether the Extreme Collaboration methodology has effectively developed the established competencies.

### Relationship between academic ambits of the capstone project and ABET and CDIO criteria

The competencies and skills in the Capstone Project were evaluated according to the CDIO learning objectives and the ABET requirements. Both CDIO and ABET initiatives summarize a set of skills, attitudes, and knowledge that students should have in their training, including those of a professional nature (leadership, communication, teamwork, and understanding of ethics and professional problems) within a global and social context, promoting learning and knowledge of contemporary issues necessary for the practice of modern engineering.

Subsequently, based on several previous investigations, eight academic aspects and their relationship with the criteria established by ABET and CDIO were selected, as shown in Table 3. Then, using Table 4, academic skills and relationships through bibliographic review were corroborated. Using Tables 3 and 4 as an analysis tool was essential to establish a clear connection between theory and practice. By contrasting the literature review results with the ABET and CDIO criteria, the validity and reliability of the methodology used could be corroborated. This methodological approach can be replicated in future research to evaluate the impact of different pedagogical interventions on developing engineering skills.

**Table 3:** Relationship between academic ambits and ABET and CDIO criteria.

No.	ABET, CDIO	^73^	^74^	^75^	^76^	^77^	^78^	^29^	^79^	^80^	^81^	^57^	^1^	^68^	^82^
1	SO2, SL2	**✓**			**✓**	**✓**	**✓**		**✓**			**✓**		**✓**	
2	SL2	**✓**	**✓**	**✓**	**✓**	**✓**	**✓**	**✓**	**✓**	**✓**	**✓**	**✓**	**✓**	**✓**	**✓**
3	SO1, SL2		**✓**		**✓**	**✓**	**✓**	**✓**		**✓**	**✓**	**✓**			**✓**
4	SO1, SL2					**✓**			**✓**	**✓**	**✓**	**✓**			
5	SO7, SL1				**✓**	**✓**				**✓**	**✓**	**✓**			**✓**
6	SO6, SL1	**✓**	**✓**	**✓**	**✓**	**✓**	**✓**	**✓**	**✓**	**✓**		**✓**	**✓**	**✓**	**✓**
7	SO7, SL1		**✓**	**✓**		**✓**		**✓**		**✓**	**✓**	**✓**	**✓**	**✓**	**✓**
8	SO5, SL3	**✓**	**✓**	**✓**	**✓**	**✓**	**✓**	**✓**	**✓**	**✓**	**✓**	**✓**	**✓**	**✓**	**✓**

1 = Architectural modeling; 2 = Structural modeling; 3 = Sanitary-hydraulic modeling; 4 = Electrical modeling; 5 = Air conditioning modeling; 6 = Budget and Cost Estimation; 7 = Teamwork; 8 = Documentation and Manuals.

**Table 4:** Relationship between skills and academic ambits.

Skills and competencies	ABET, CDIO	Ambit^*^	^73^	^74^	^75^	^76^	^77^	^78^	^29^	^79^	^80^	^81^	^57^	^1^	^68^	^83^	^82^
Ability to plan and systemic thinking.	SO6, SL2	1,2,3,4,5,6,7,8	**✓**	**✓**	**✓**	**✓**	**✓**	**✓**	**✓**			**✓**	**✓**		**✓**	**✓**	**✓**
Demonstrates learning and knowledge discovery.	SL2	1,2,3,4,5,8	**✓**	**✓**		**✓**	**✓**	**✓**	**✓**				**✓**	**✓**	**✓**	**✓**	**✓**
Ability to identify complex engineering problems.	SO1, SL2	1,2,3,4,5	**✓**		**✓**			**✓**	**✓**				**✓**	**✓**	**✓**		**✓**
Ability to formulate and solve complex engineering problems.	SO1, SL2	1,2,3,4,5,7			**✓**	**✓**			**✓**			**✓**		**✓**	**✓**	**✓**	
Ability to acquire and apply new knowledge of engineering.	SO7, SL1	1,2,3,4,5,6,7,8	**✓**			**✓**		**✓**	**✓**			**✓**	**✓**		**✓**	**✓**	**✓**
Ability to analyze and interpret data.	SO6, SL1	6,8				**✓**			**✓**				**✓**	**✓**	**✓**	**✓**	
Ability to apply engineering design to produce solutions.	SO2, SL1	1,2,3,4,5	**✓**	**✓**			**✓**		**✓**	**✓**	**✓**		**✓**				
Ability to function in multidisciplinary teams and create a collaborative and inclusive environment.	SO5, SL3	7	**✓**	**✓**	**✓**	**✓**	**✓**	**✓**	**✓**	**✓**	**✓**	**✓**	**✓**	**✓**	**✓**	**✓**	**✓**
Ability to communicate effectively with a range of audiences.	SO3, SL3	7,8		**✓**		**✓**	**✓**					**✓**	**✓**	**✓**		**✓**	**✓**
It conceives and generates required (Plans, technical specifications, budget).	SL4	1,2,3,4,5,6,8	**✓**		**✓**			**✓**	**✓**					**✓**	**✓**	**✓**	**✓**
Ability to recognize ethical and professional responsibilities in engineering.	SO4, SL2	1,2,3,4,5,6,7,8		**✓**	**✓**	**✓**			**✓**		**✓**			**✓**		**✓**	**✓**

*1 = Architectural modeling; 2 = Structural modeling; 3 = Sanitary-hydraulic modeling; 4 = Electrical modeling; 5 = Air conditioning modeling; 6 = Budget and Cost Estimation; 7 = Teamwork; 8 = Documentation and Manuals.

**Fig. 3:**
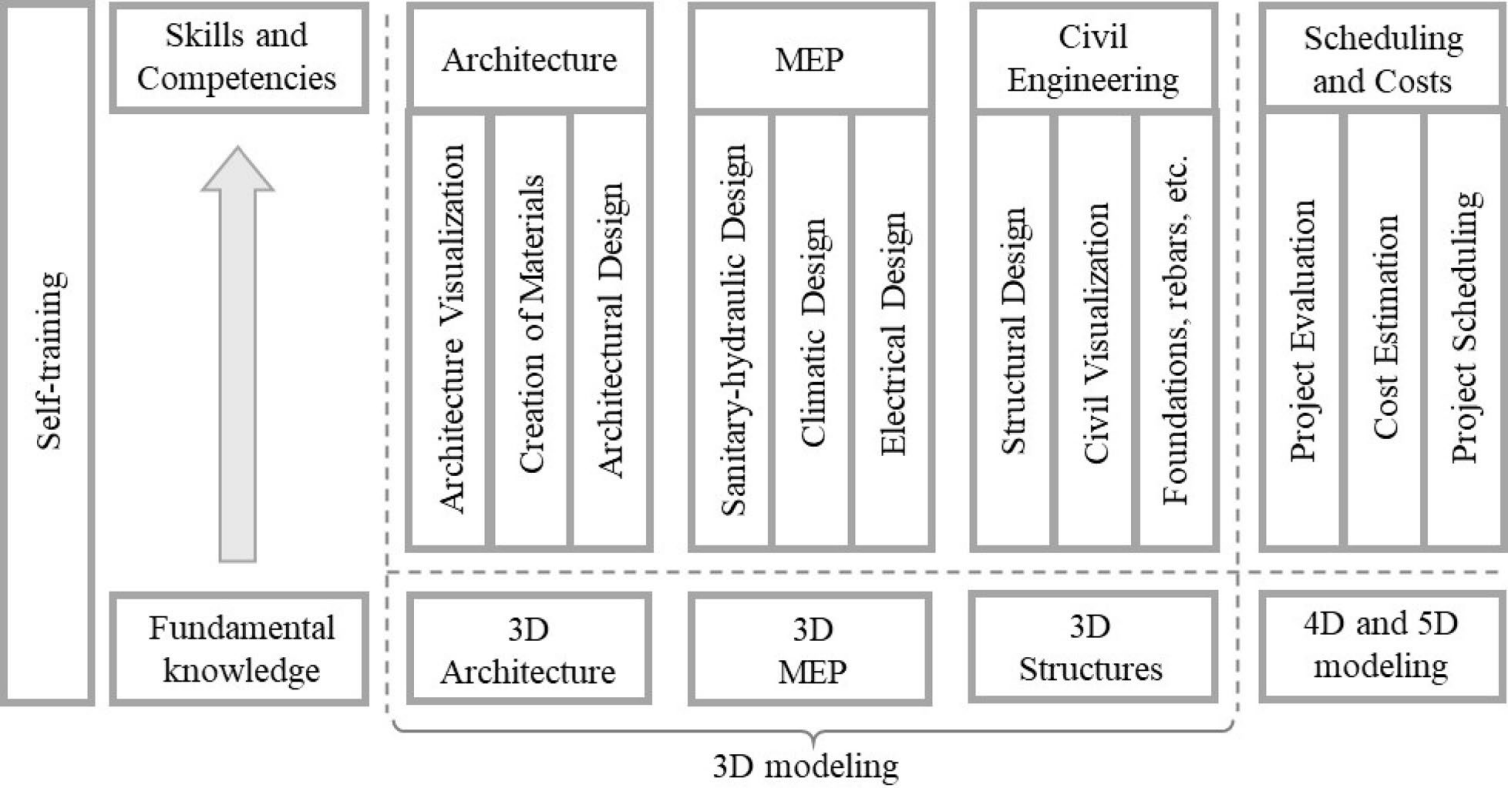
Abilities and capabilities acquired by the students in terms of the activities developed.

Finally, Fig. 3 visually represents how students develop their skills and competencies throughout the design process, from basic knowledge to more advanced applications. The figure is divided into two main sections: (1) Fundamental Knowledge: This section represents the foundation on which more specific skills are built. It includes essential theoretical and practical knowledge in various areas such as architecture, mechanical, electrical, and civil engineering; and (2) Skills and Competencies: This section shows how students apply fundamental knowledge through different tools and software to develop specific projects. It is divided into four main areas: Architecture, MEP (mechanical, electrical, plumbing), civil engineering, scheduling, and costing. It highlights the wide range of skills that students can acquire through the design process, from technical skills such as the use of specialized software (Revit) to more general skills such as visualization and problem-solving and the inclusion of different areas of engineering (architecture, MEP, civil) reflects the multidisciplinary nature of many design projects today. As with the architectural design phase —where tools such as SketchUp or ArchiCAD can serve as alternatives to Revit Architecture— the tools used for scheduling and cost management may vary across projects. In this study, the “Scheduling and Costs” component shown in Fig. 3 refers to the integrated implementation of 4D and 5D BIM processes rather than a single software application. Specifically, the 4D scheduling was developed using Microsoft Project, which was integrated with Revit through Navisworks to simulate construction sequencing and visualize the project progress. For cost estimation (5D), Excel spreadsheets were used to manage and track costs linked to model elements. In terms of project evaluation, the chosen combination reflects an interoperable workflow between commonly used software platforms, where Revit, Navisworks, MS Project, and Excel enabled the review of the 3D model, along with its schedule (4D) and cost (5D). Therefore, the label “Scheduling and Costs” is intended to represent the functional integration of these BIM dimensions (4D and 5D), as a complement to the 3D modeling.

Therefore, it is evident that the design process promotes a comprehensive acquisition of skills in students^4,84–87^. By applying theoretical knowledge to practical projects and using specialized tools such as Revit, students develop technical and soft skills essential for the professional field. Likewise, the collaborative nature of design projects encourages critical thinking, problem-solving, and teamwork, preparing future professionals to face the challenges of an increasingly complex work environment.

## Methodology

The present study was designed as an exploratory investigation. Exploratory research plays a significant role in the development of new knowledge, particularly in fields where established theories or benchmarks are not yet well-defined, such as the XC methodology. An exploratory design can provide foundational insights that guide and shape future investigative efforts, as it allows researchers to become familiar with evolving phenomena and to propose new frameworks or understandings. For example, in the context of pedagogical research, exploratory studies can reveal overlooked aspects of teaching and learning that more rigid, hypothesis-driven designs might miss^88,89^. They also offer a flexible way to examine complex social or psychological behaviors in real-world settings^90,91^. While it’s true that the absence of a control group limits some types of comparisons, the insights gained through this methodology can lay essential groundwork for future, more controlled research. In fields such as engineering education, where innovation often outpaces theoretical development, this type of foundational inquiry is a crucial component of the research process^92^.

Thus, the research design employed in this study is exploratory and descriptive in nature, utilizing a single-group quasi-experimental approach with implicit pre- and post-assessments. It is exploratory in that it seeks to understand the implementation of a relatively novel (XC) methodology in a specific context (a civil engineering Capstone project). It is descriptive in that it meticulously details the phases of the procedure and the observed outcomes in terms of competency development. The absence of a control group of students who did not receive the XC intervention classifies it as a single-group design, where the comparison is implicitly made against the standards of required professional skills and the expectations of expert professionals in engineering education, as mentioned above.

The reasons for this type of design focus on the depth of analysis regarding the application of the XC methodology and its impact on a select and representative group of students (a purposive sample of six students with outstanding performance and specific skills). This approach enables a detailed examination of the collaborative process, BIM integration, and competency assessment, based on Bloom’s Taxonomy and ABET/CDIO frameworks, providing rich insights into how the experience unfolds and its direct outcomes for participants.

Regarding informed consent for subjects participating in this research, the students voluntarily agreed to take part in the study and were provided with information about its purpose, procedures, potential risks, and benefits. They understood that their participation was entirely voluntary and that they could withdraw at any time without penalty or loss of benefits. The students had the opportunity to ask questions about the study, and all inquiries were answered to their satisfaction.

Additionally, all methods used in this research were conducted in strict adherence to relevant ethical guidelines and regulations, including University Decree No. 2070 − 2024 on the Regulations of the Scientific Ethics Committee of the University of Bío-Bío, Chile.

All experimental protocols utilized in the Extreme Collaboration Laboratory outlined in this project underwent rigorous review and approval by the Scientific Ethics Committee of the University of Bío-Bío, Chile. This committee consists of experts in safety, ethics, and technology, who evaluated the safety, feasibility, and ethical compliance of each experimental protocol prior to implementation.

Each participant’s role was defined to implement the XC methodology in the Capstone Project. Subsequently, the students became familiar with the methodology and met their teammates. Then, before beginning the learning process, they studied the project and its constructive details. Finally, the students proceeded to the 3D modeling process, where the results, shown below, were evaluated.

### Methodology of a capstone project

The methodology associated with a Capstone Project course begins with sessions that explain the objectives, what the course will consist of, and how it will be developed, including the steps for correct planning and resolution of the student’s doubts^93–95^. Subsequently, it defines the projects to be developed and their respective groups of students, and then discusses each with the course guides^96^ through presentations, reports, and talks. The project design process begins with meetings with professors and students to review progress, and therefore, students can make inquiries and clarify doubts, yielding discussions about the problems encountered^33,95,97^. Finally, through final presentations, the students deliver and explain the achievement of the proposed goals through drawings, technical specifications, budgets, and reports^15,33,86^.

### Description of the collaborative process

**Fig. 4:**
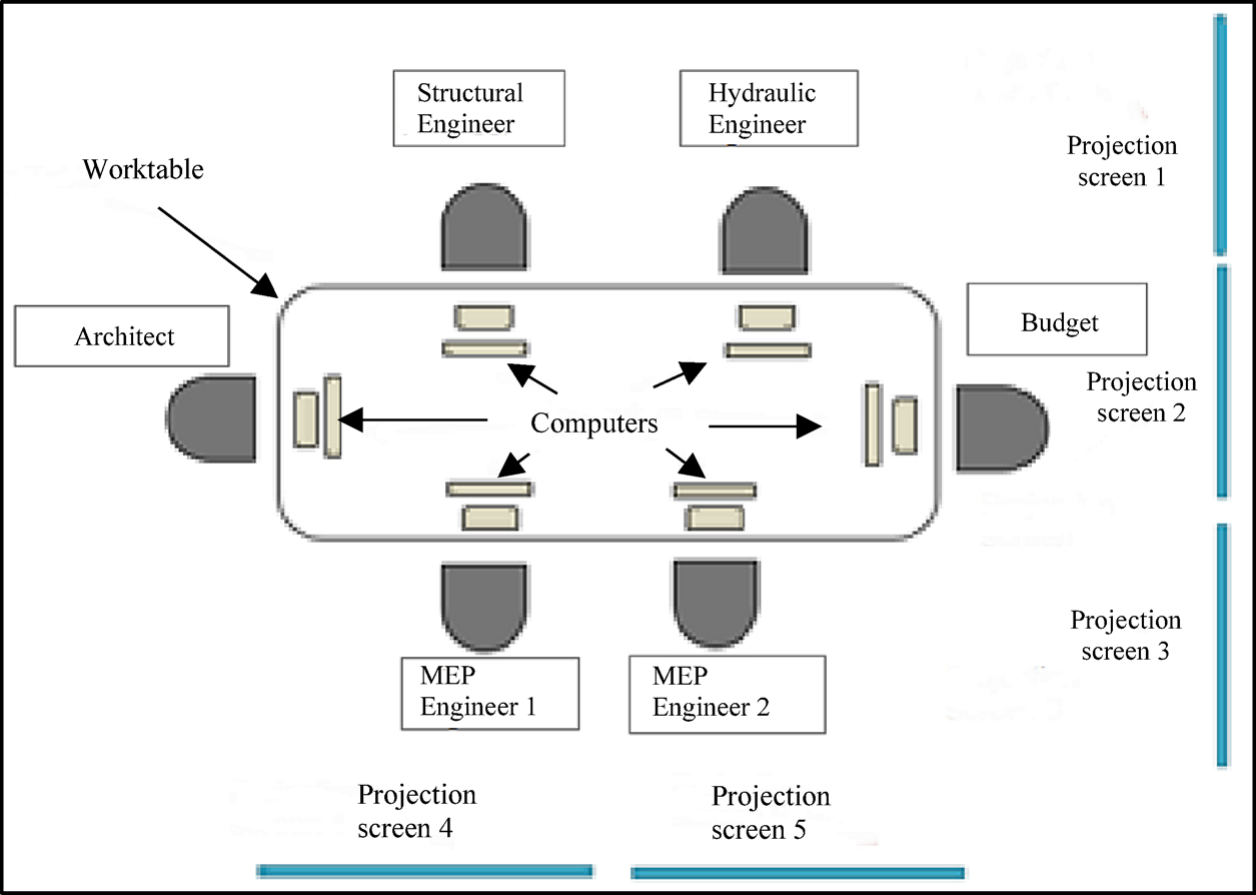
Schematic representation of the Integrated Project Design Laboratory, where the XC experience was applied to Capstone Project course students.

**Fig. 5:**
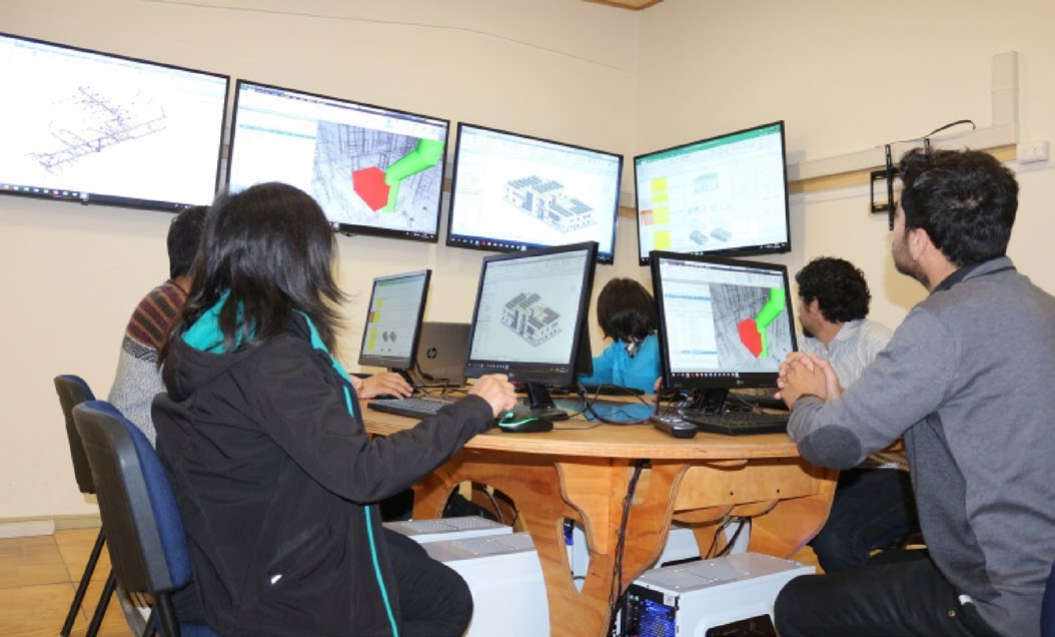
Capstone Project session under the XC methodology, in the Integrated Project Design Laboratory.

A purposive sample of six students was selected to participate in the Capstone Project of the Civil Engineering program at a university in southern Chile. The selection was based on specific criteria: outstanding academic performance in engineering project courses, a demonstrated interest in research, and the ability to showcase skills relevant to the study, such as teamwork and problem-solving. Although purposive sampling does not aim for statistical representativeness of the student population, these criteria were employed to ensure that participants possessed the essential competencies to engage meaningfully in research and maximize the richness of the qualitative data generated. While exploratory research does not require a predetermined minimum sample size^98^the total number of eligible students in the Civil Engineering program who met the initial criteria for consideration in the purposive selection was 25 students, where the six participants selected accounted for 24% of the eligible population. The methodological process was carried out in the Integrated Project Design Laboratory of the same university, as illustrated in Fig. 4 (Schematic representation of the Integrated Project Design Laboratory) and Fig. 5 (Capstone Project sessions using the XC methodology).

The students participating in the Capstone Project course met three times a week, for three-hour sessions each, over four months, similar to previous studies reviewed in the literature review^40,73,86^. This period resulted in a total of 17 work sessions, all developed under the framework of the Extreme Collaboration Methodology. This methodology, characterized by its highly collaborative and dynamic approach, allowed the students to work together and intensively on their final projects. The characteristics of the methods used are shown in Table 5.

**Table 5:** Description of the XC methodology used in the Capstone Project.

Item	Description
Number of participants	6 Participants per session
Place	Integrated Design Laboratory
Hours per session	3 h
Number of sessions per week	3 Sessions per week
Number of screens projected	4 Screen projected
Number of participants per worktable	6 Participants per worktable
Number of worktables	1 Worktable
Specialists who make up the team	Budget Manager (1)
	Structural Project Manager (1)
	Architectural Project Manager (1)
	MEP Project Managers: Mechanical (1); Electrical (1); Plumbing (1). In total, 3

### Project description

The project used to carry out the Capstone Project course under an XC methodology corresponded to a two-story structure with a total area of 277.53 m^2^whose characteristics are shown in Table 6.

**Table 6:** Project characteristics.

Item	Characteristics
N° of floors	2
Total area	277.53 m^2^
N° of offices	10
N° of bathrooms	2
Specialties	Architecture Structures Hydraulic and sanitary Electricity Air conditioning

### Procedure

The procedure used for making the experience consisted of four phases. The first two stages help to understand the project and emphasize the importance of integrated design and collaborative work. The design and evaluation process were carried out in the following two stages. Table 7 shows the stages and duration of each of them and their subsequent explanation.

**Table 7:**
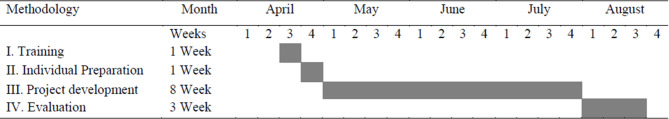
Gantt chart of the working methodology used in the study.

On the other hand, to assess the technical performance of students using an XC methodology, through a technique of systematic observation and continuous recording of the specific events that occurred during the 17 work sessions of the Capstone Project, the data collected by each specialty was analyzed by counting the number of inconsistencies in the 3D model during each session. These inconsistencies arise from interactions between the specialties involved in the Capstone Project (Architecture, Structures, Hydraulics, Electricity, Climate). Similarly, the number of personal conflicts within each specialty per session was also tallied and analyzed. The hypothesis was that increased interaction among students would lead to fewer inconsistencies and conflicts during the Capstone project development. The analysis, which confirmed this hypothesis, is presented later in the Results section (Figs. 8 and 9).

**Fig. 6:**
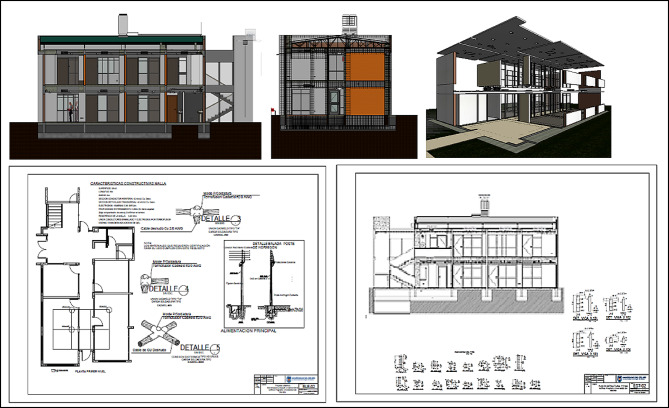
Model Design in Extreme Collaboration.

**Fig. 7:**
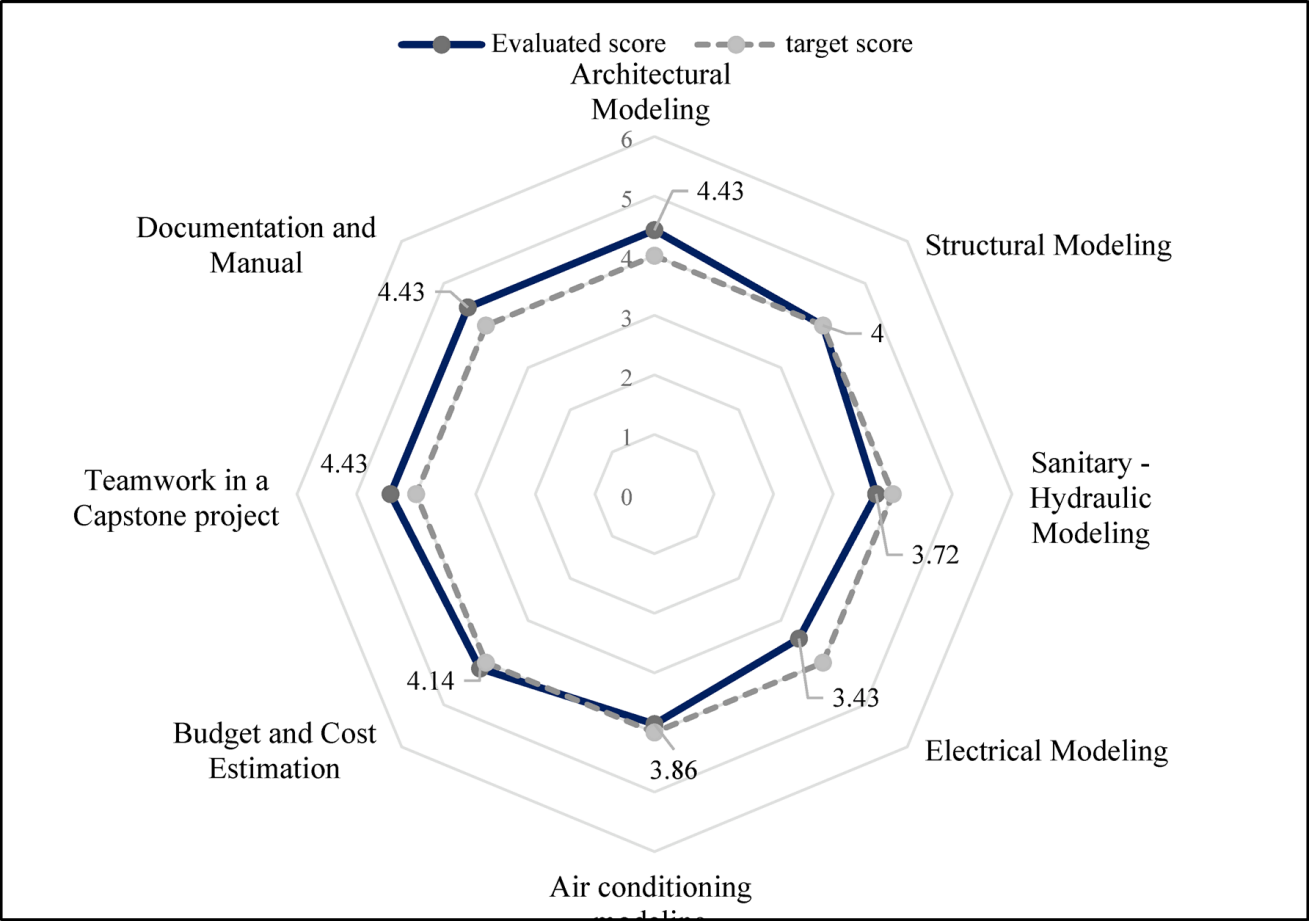
Progress level acquired by the students – Mean of the domains evaluated.

### Stage I: training

The participants were trained in BIM, which is used as a methodology for developing collaborative and integral works in the construction industry, achieving adequate and efficient project development^99–101^. Subsequently, roles were assigned to each one, and drawings and technical specifications were studied so that they could understand each specialty. Finally, each student was trained in his/her specialty to achieve optimal modeling during the design process. Given the importance of collaborative work, the research scope, and the course’s objectives, the classes were guided by experts and users with experience in synchronized modeling. BIM training, combined with the assignment of specific roles and the detailed study of drawings and specifications, enabled participants to develop the skills necessary to model their respective specialties optimally. Guidance from experts and users with experience in synchronized modeling was essential to ensure practical collaborative work and the achievement of the course objectives. The results of previous studies demonstrate that the implementation of BIM in the training of future construction professionals is an effective strategy to promote collaboration, efficiency, and quality in the development of projects^86,102–105^.

### Stage II: individual Preparation

Individual preparation is essential to ensure students understand the required skills and capabilities and have information about their disciplines. That is why they met with experts and evaluators with more than 10 years of experience who reviewed the project documentation (technical specifications, drawings, and bidding documents), facilitating the understanding of the project and the tasks. By providing access to experts and detailed technical documentation, participants gained a deep understanding of the project and the skills required for its development^86,100,105^. This stage demonstrates that solid and specific preparation is essential to ensure success in collaborative projects.

### Stage III: project development

The students developed the project using the XC methodology. MEP’s architectural, structural, and components (Mechanical, Electrical, and Plumbing) were created at this stage. Implementing the Extreme Collaboration methodology allowed students to develop a comprehensive architectural project, from initial conception to the production of complete technical documentation. Creating detailed models like those in Fig. 6 demonstrates students’ ability to work collaboratively and produce high-quality results in a multidisciplinary design environment. The documents generated, including reports, technical specifications, and cost estimates, provide a solid basis for the execution of the project.

**Fig. 8:**
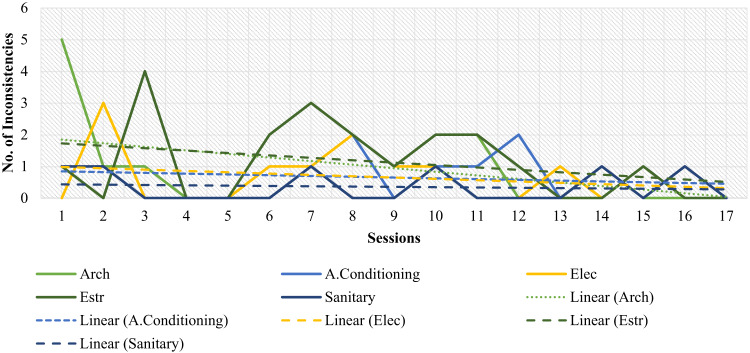
Inconsistencies of specialties by session.

### Stage IV: project evaluation

Educators evaluated the project according to the results in the final part of the project development. Students presented drawings, schedules, technical specifications, cost estimates, and reports. The fulfillment of the abilities and capacities of the individuals is evaluated according to the set of skills and competencies proposed by ABET and CDIO (as previously shown in Table 1). This research shows the importance of evaluating projects thoroughly, considering the final products and the development process. Aligning the evaluation with international standards such as ABET and CDIO guarantees the quality of training and the relevance of the knowledge acquired by students. The results obtained are promising and open new perspectives for improving teaching and learning processes in engineering.

## Results

A monitoring and evaluation process was carried out to assess the development of students’ academic skills. This process included direct observation of the work sessions and a detailed review of the plans, reports, and other documents prepared by the students. The results obtained were compared with Table 4, which serves as a reference to determine whether the skills developed by the students meet the international ABET and CDIO standards. Based on the rubric previously shown in Table 2, the evaluation of the academic domains was conducted, and Table 8 shows the academic domains and the corresponding percentages related to the student’s performance in Bloom’s taxonomy (1 to 6).

In this context, Table 8 shows the assessment results for the academic domains as percentages. These values are directly based on the rubric outlined in Table 2, which aligns with Bloom’s Taxonomy. For each of the eight areas (such as Architectural modeling, Structural modeling, etc.), the evaluators —professors responsible for the Capstone project course— used specific criteria defined for each of the six Bloom levels (Remember, Understand, Apply, Analyze, Evaluate, Create) in the rubric displayed in Table 2. As a result, the percentages in Table 8 indicate the proportion of the six students who reached or exceeded a particular cognitive level for each skill. For instance, if in the academic domain “Architectural modeling” 42.9% of students are at the “Evaluate” level (5), as shown in Table 8, it means that this percentage of students demonstrated the ability to evaluate solutions per the criteria listed in the “Evaluate” column of the rubric in Table 2. This rubric thus served as a standardized measurement tool, enabling the quantification of students’ progress across different levels of thinking and providing a detailed view of how the XC methodology impacted their competency development.

**Table 8:** Results of the evaluation of academic areas (in percentages).

Academic domains	(1)	(2)	(3)	(4)	(5)	(6)
Architectural modeling	-	-	28.6	14.3	42.9	14.3
Structural modeling	-	-	42.9	28.6	14.3	14.3
Sanitary-hydraulic modeling	-	14.3	42.9	14.3	14.3	14.3
Electrical modeling	-	14.3	57.1	14.3	-	14.3
Air conditioning modeling	-	14.3	42.9	14.3	-	28.6
Budget and Cost estimation	-	14.3	-	57.1	14.3	14.3
Teamwork	-	14.3	-	42.9	14.3	28.6
Documentation and Manual	-	-	14.3	57.1	-	28.6

Note: 1 = Remember; 2 = Understand; 3 = Apply; 4 = Analyze; 5 = Evaluate; 6 = Create.

**Fig. 9:**
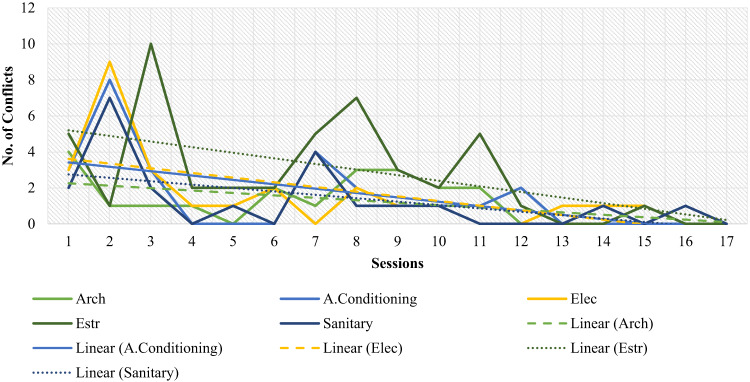
Conflicts of specialties by session.

The scores obtained for each academic area were averaged according to Eq. 1, and the results are shown in Fig. 9.

1 $$\:P=\frac{\sum\:_{i=1}^{n}B\bullet\:S}{100}$$

Where *P* is the average level of student progress, *B* is Bloom’s taxonomy level, *S* is the students’ score in percentage obtained after evaluation, and *n* is the number of taxonomy levels (6). For example, the following calculations illustrate how the average progress level, P, was determined for the Sanitary-Hydraulic modeling academic domain, using the information presented in Table 8. The same calculation was applied to all the academic domains displayed in Fig. 7.

$$\:P=\frac{\sum\:_{i=1}^{n}B\bullet\:S}{100}=\frac{1\bullet\:0+2\bullet\:14.3+3\bullet\:42.9+4\bullet\:14.3+5\bullet\:14.3+6\bullet\:14.3}{100}=3.72$$

Thus, as Bloom’s taxonomy searches for evaluating different levels of progress of the student’s cognitive domain in the learning process, Table 8 shows that no students obtained the lowest value (column 2, level of progress 1, Remember). Although this result might be seen as a potential issue, since participants cannot progress without basic knowledge, it actually highlights the effectiveness of the individual training and preparation phase. It shows that the intensive BIM training and specialized preparation, combined with role assignment and expert guidance (Stages I and II), were so successful that students quickly moved beyond the basic “Remember” level. This outcome indicates that the XC methodology not only helped with information retention but also directly pushed students to higher cognitive levels, such as “Understand,” “Apply,” and “Analyze,” from the start of the project, demonstrating a deep and active grasp of knowledge essential for developing a complex Capstone project. On the other hand, most students obtained values higher than level 3 (Apply), and mainly concentrated between levels 4, 5, and 6 (Analyze, Evaluate, and Create). In other words, the average performance of students in all areas evaluated exceeded the “application” level (level 3) of Bloom’s taxonomy. This finding is significant, given that previous research has highlighted the importance of transcending mere knowledge toward the practical application of concepts in the cognitive process. In contrast to other areas, notable progress is observed at the “Analysis” level (Level 4 in Bloom’s Taxonomy), surpassing five of the eight academic domains assessed in Fig. 7. Specifically, in sanitary-hydraulic modeling (3.72), electrical modeling (3.43), and air conditioning modeling (3.86), students achieved average scores that are consistently established between the “Apply” (3) and “Analyze” (4) levels. This demonstrates the students’ ability to go beyond the mere application of knowledge, managing to break down information and establish complex relationships, which is crucial for problem solving in civil engineering.

Furthermore, to analyze student performance, as mentioned in the Methodology section, through systematic observation and continuous recording of specific events during the 17 sessions of the Capstone project using the XC methodology, the number of inconsistencies between the project’s specialties and the number of conflicts between students (misunderstandings, technical interpretation issues, or poor delivery of information) were analyzed. The data collected by specialty in each session were plotted in Figs. 8 and 9, respectively, and then analyzed. Specifically, inconsistencies in the 3D model (Fig. 8) were identified and quantified through the review and audit of the BIM model in each session, recording interferences or clashes between components from different specialties (Architecture, Structures, Hydraulics, Electrical, and Climate) during the integration of their models. In parallel, the number of personal conflicts (Fig. 9) was documented through direct observation of student interactions by the researchers and evaluators present, who classified and counted disagreements or differences in interpretation that arose during the collaborative process. This review not only quantified the frequency of these events over time but also correlated their occurrence with the development of competencies, demonstrating how the XC methodology, by generating these “stimuli” for ongoing interaction, promoted the practice of interpersonal and problem-solving skills.

As hypothesized, as the Capstone Project evolved, variations in both interferences and conflicts tended to stabilize and decrease over time, as shown in Figs. 8 and 9. This finding could provide some evidence that the permanent interaction demanded by the XC methodology might be beneficial in reducing interferences and conflicts in a Capstone Project.

Throughout the process, interferences arose in 3D modeling, as well as conflicts of both anecdotal and academic nature. Each of the conflicts provoked in the participants stimulated them to put their competencies into practice. Some disciplines performed less than expected due to little interaction and activity throughout the process. In Fig. 8, the trend line between disciplines is observed. Those with lower qualifications show that their trend line is of a lower slope; that is, they did not make significant improvements. Such specialties can be promoted by developing targeted areas, creating action plans, and providing guidance.

**Fig. 10:**
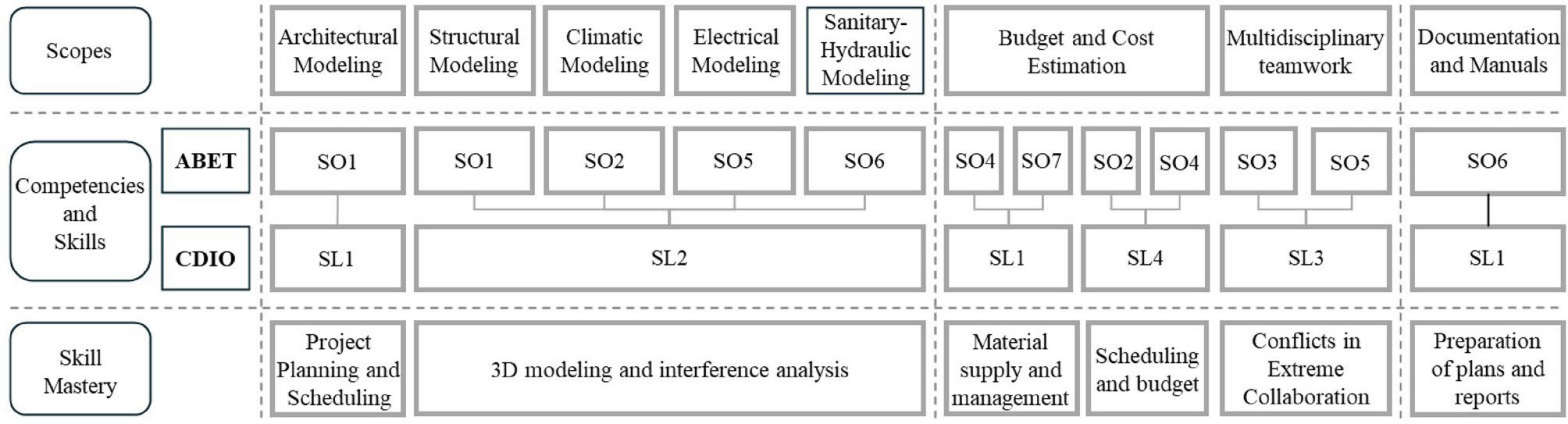
Relationship of specialized knowledge, competencies, and skills.

Finally, the competencies proposed by ABET and CDIO were implemented during the course development. Figure 10 shows —through a conceptual map— the capabilities and skills that were put into practice through diverse activities and conflicts generated in the evaluation process.

## Discussion

Through the experience, it was possible to appreciate the improvements found in the Capstone Project by implementing a work methodology based on collaboration, where the students worked in three days-a-week sessions with three hours of work each session. The project was optimally developed (working time and its objectives were as expected), although it emphasized the stages of training and individual preparation of students, achieving at the time of modeling. The proposal consisted of carrying out the training stages in two weeks and the individual preparation in two others because they play a fundamental role in obtaining skills. The participants were trained in the fundamental knowledge of their corresponding specialties, and they were informed about the methodology implemented. The proposal should not increase working time but optimize it.

Recommendations have also been provided for structuring engineering programs around the CDIO model, highlighting the relevance of conceptualizing, designing, implementing and operating, and focusing on the construction and maintenance of the delivered civil engineering solutions. According to the authors, the presented XC methodology —reformulated here for civil engineering— contributes to most of the CDIO curriculum outcomes and ABET quality assurance guidelines. Furthermore, the provided basic architecture and engineering of a complete building and its subsystems, supported by BIM methods under a collaborative methodology, immerses students in a real-life scenario and significantly contributes to their professional development, reinforcing technical and soft skills.

During this evaluation process, students were observed to practice each of the skills and capabilities proposed by CDIO and ABET. For example, in Figs. 8 and 9, it can be distinguished that in sessions 3, 7, and 11, there is a direct relationship between inconsistencies and the number of conflicts. That is why, whenever there was interference or a conflict, students were encouraged to put into practice some of the competencies proposed by ABET and CDIO, such as interpersonal skills, teamwork and communication (SL3), ability to function as a multidisciplinary team (SO4), the ability to identify, formulate and solve engineering problems (SO5), among others.

In the specialty of structures, it is observed that it had an active participation, generating conflicts in the design of this specialty, evidencing the practice of different skills in the design process such as applying engineering knowledge (SO1), ability to design a system under different restrictions (SO3), ability to analyze and interpret data (SO2). Moreover, others such as disciplinary knowledge and reasoning (SL1), experimentation, research and systems thinking (SL2), and the ability to conceive, manage, and design systems (SL4). In general, the active participation of each specialty in the evaluation process can be seen, evidencing their teamwork (SO4).

On the one hand, in an open question made to the participants in the project, they indicated certain elements that they considered extremely important in the first phases of the project: individual training and preparation to promote fundamental knowledge. In the first phase of the project (training), the students also noted that they could be given more emphasis and preparation time. Thus, the evaluators concluded that students learn more from their assigned specialties than from others through constant interaction with their peers in collaborative work. Even so, the evaluators found that the experience improved the disciplines’ performance through action plans and guidance.

Another advantage of XC in civil engineering is that it constitutes a resource-efficient teaching-learning experience. Furthermore, it can be straightforwardly replicated even in emergencies like those living in the recent COVID-19 pandemic. Indeed, XC in civil engineering can be developed as a fully remote educational experience involving delocalized teams of international engineers that work together from their study places, even in lockdown situations.

Among the potential limitations that the application of the XC methodology could have in the university setting is the need for infrastructure, which, although it has a moderate cost (US$20,000 approximately to equip an integrated project design laboratory), could be relevant for massive applications of the methodology, primarily if it is intended to extend its application to a more significant number of civil engineering students throughout the curricula and even to other engineering specialties. Another difficulty is that XC is not used massively by companies worldwide, which could result from a lack of knowledge, the infrastructure requirement, or the existence of alternative methodologies, such as agile methodologies.

In this context, a line of future research is the comparison of XC with agile methodologies, such as Scrum or Lean Construction, which are more widely used in the business environment and higher education. This comparison would allow determining, for example, under what conditions XC would be preferable, which could favor the transfer of this methodology from the academy to the industry.

### Theoretical contribution

This research assessed the impact of XC on developing professional competencies in a civil engineering Capstone project, integrating BIM and Bloom’s Taxonomy. Findings show XC is an effective teaching approach. Evaluation of skills at higher Bloom’s levels, along with the reduction in 3D model inconsistencies and conflicts, addresses the goal of bridging the gap between academic training and professional demands. Results confirm that XC helps students develop both technical skills in BIM and soft skills, such as teamwork, problem-solving, and communication, which are vital for 21st-century engineering.

Comparing these results with existing literature shows a strong similarity to studies supporting active and collaborative teaching methods for developing engineering skills^106–108^. The clear integration of BIM and Bloom’s Taxonomy, along with detailed tracking of inconsistencies and conflicts, offers a more detailed and measurable view of collaboration’s impact compared to broader research on project design^109^. While the literature recognizes the importance of project-based learning^110,111^this study reinforces and expands that view by showing how structured methods like XC can tackle collaboration challenges and boost competencies defined by ABET and CDIO, improving professional readiness.

The findings highlight that implementing the XC methodology in Capstone projects or design courses is crucial for preparing engineers for real-world challenges. Educators should shift to facilitation, creating environments that mimic engineering complexity and promote collaboration. Institutions need to invest in training and infrastructure, like integrated design labs. Ultimately, the adoption of XC can produce graduates with enhanced problem-solving, communication, and adaptability skills for a changing workplace.

### Implications for practitioners

Implementing the comprehensive XC methodology in civil engineering Capstone projects can be both transformative and challenging. Educators must manage students’ expectations, as students used to traditional teaching may resist the autonomy of XC. A strong introductory phase, highlighting the benefits of collaboration and offering soft skills workshops, is essential. Adequate technological resources and collaborative spaces, such as the Integrated Project Design Lab, are necessary. Limited environments require optimizing resources, using online tools, and remote sessions. Assessing performance in a collaborative setting can be challenging; detailed rubrics that evaluate outcomes, contributions, and skills development are recommended. Ongoing feedback and reflection help students adjust to their learning. Anticipating challenges and providing solutions can enhance the effectiveness of approaches like XC, preparing engineers for collaborative and adaptable environments.

This research is highly relevant for university engineering educators, particularly those leading Capstone courses or final projects, as well as for curriculum designers and policymakers updating engineering education. The study demonstrates that the XC methodology effectively promotes skills such as teamwork, problem-solving, and communication—key for professional practice, as per frameworks like ABET and CDIO—providing a practical guide to enhance teaching and learning. By adopting XC and tools like BIM and Bloom’s Taxonomy, educators can create experiences that teach theory and develop soft and technical skills, preparing students for 21st-century challenges. This approach bridges the gap between academic training and labor market needs.

## Conclusions

Although authors have conducted a comprehensive literature review to evaluate the effectiveness of a Capstone Project course, there is limited evidence of evaluating ABET and CDIO competencies in a Capstone Project course implemented under a collaborative methodology such as XC. CDIO and ABET aim to outline a set of skills and knowledge that students should acquire during their academic training, encompassing professional competencies, to prepare engineers for modern engineering. For this reason, compliance with the competencies proposed by ABET and CDIO in developing the Capstone Project was evaluated. The evaluation was based on Bloom’s Taxonomy, which allowed the cognitive level attained to be measured. Once the project was completed, the fulfillment of the established competencies was analyzed and evaluated by a team that supervised the project’s development and graded the documents, drawings, and reports prepared by the participants.

The results obtained in this research point out the benefits that the development of an engineering project implemented under the XC methodology brings. The research also shows that implementing a Capstone Project course using this methodology could benefit students’ academic training because it stimulates the development of professional skills through conflicts and peer interactions in a synchronized modeling process.

This study has shown that implementing a Capstone Project course under the XC methodology effectively promotes the development of ABET and CDIO skills in engineering students. Through an evaluation based on Bloom’s Taxonomy, it has been demonstrated that projects developed under this methodology enable students to reach higher cognitive levels and apply the knowledge they have acquired in real-world situations. The results support the idea that the XC methodology —by promoting collaboration and problem-solving in a simulated environment— constitutes a valuable tool for the comprehensive training of engineers.

Regarding the limitations of this study, although the effectiveness of the XC methodology in developing ABET and CDIO skills has been demonstrated, further research is needed to examine these skills over time. Long-term follow-up studies could show how skills gained in the Capstone Project are applied and solidified in graduates’ professional performance. In other words, the intentional sample of six students, selected to maximize qualitative data, limits how broadly the findings can be applied. Future research should include larger samples and a control group to better establish causality. Exploring graduates’ long-term perceptions and comparing XC with other active learning methods across various settings would be helpful. Investigating how factors like team size, diversity, and project type influence outcomes is important. Gathering students’ perceptions of XC’s impact on professional growth and satisfaction would add value. Developing specific teaching strategies to strengthen underdeveloped skills and improve Capstone Project courses is also recommended.

## Data Availability

Data sets generated during the current study are available from the corresponding author upon reasonable request.
